# The Role of Sensor-Activated Faucets in Surgical Handwashing Environment as a Reservoir of *Legionella*

**DOI:** 10.3390/pathogens9060446

**Published:** 2020-06-05

**Authors:** Marta Mazzotta, Luna Girolamini, Maria Rosaria Pascale, Jessica Lizzadro, Silvano Salaris, Ada Dormi, Sandra Cristino

**Affiliations:** 1 Department of Biological, Geological, and Environmental Sciences, University of Bologna, via San Giacomo 12, 40126 Bologna, Italy; marta.mazzotta2@unibo.it (M.M.); luna.girolamini2@unibo.it (L.G.); mariarosaria.pascal2@unibo.it (M.R.P.); jessica.lizzadro2@unibo.it (J.L.); silvano.salaris@unibo.it (S.S.); 2 Department of Medical and Surgical Science, University of Bologna, via San Giacomo 12, 40126 Bologna, Italy; ada.dormi@unibo.it

**Keywords:** Healthcare-Associated Infections (HAIs), Surgical Handwashing Outlets (SHWOs), sensor-activated faucets, *Legionella* spp., risk assessment plan

## Abstract

Surgical handwashing is a mandatory practice to protect both surgeons and patients in order to control Healthcare-Associated Infections (HAIs). The study is focused on *Legionella* and *Pseudomonas aeruginosa* contamination in Surgical Handwashing Outlets (SHWOs) provided by sensor-activated faucets with Thermostatic Mixer Valves (TMVs), as correlated to temperature, technologies, and disinfection used. Samples were analyzed by standard culture techniques, comparing hot- and cold-water samples. *Legionella* isolates were typed by an agglutination test and by *mip* sequencing. *Legionella* contamination showed the same distribution between hot and cold samples concerning positive samples and mean concentration: 44.5% and 1.94 Log_10_ cfu/L vs. 42.6% and 1.81 Log_10_ cfu/L, respectively. Regarding the distribution of isolates (*Legionella pneumophila* vs. *Legionella* non-*pneumophila* species), significant differences were found between hot- and cold-positive samples. The contamination found in relation to ranges of temperature showed the main positive samples (47.1%) between 45.1–49.6 °C, corresponding to high *Legionella* concentrations (2.17 Log_10_ cfu/L). In contrast, an increase of temperature (>49.6 °C) led to a decrease in positive samples (23.2%) and mean concentration (1.64 Log_10_ cfu/L). A low level of *Pseudomonas aeruginosa* was found. For SHWOs located in critical areas, lack of consideration of technologies used and uncorrected disinfection protocols may lead to the development of a high-risk environment for both patients and surgeons.

## 1. Introduction

Nosocomial infections, also known as Healthcare-Associated Infections (HAIs), are defined as infections which were absent at the time of hospital admission that a patient acquires during their stay in a hospital or other healthcare facilities [1]. Populations that are at risk for HAIs are immunocompromised patients in Intensive Care Units (ICUs), those in burn units, those undergoing organ transplants, or older patients and neonates. Extensive studies have been carried out by the World Health Organization (WHO) showing that the most frequent nosocomial infections globally include catheter-associated urinary tract infections, central-line associated bloodstream infections, ventilator-associated pneumonia, and surgical site infections [1].

It has been estimated that, in Italy, 5–8% of hospitalized patients contract nosocomial infections every year and 450,000–700,000 HAIs occur in hospitalized patients; these data refer to urinary infections, followed by infections of surgical wounds, pneumonia, and sepsis [2,3].

Risk factors that promote nosocomial infections—other than patient susceptibility, such as immunosuppressed patients in ICUs—include poor hygienic conditions such as improper hand hygiene of Healthcare Staff (HCS) or contaminated air and water [1]. The water supply system in hospitals may constitute a source of HAIs caused by opportunistic pathogens such as *Pseudomonas aeruginosa* (*P. aeruginosa*), *Legionella* spp., *Acinetobacter* species, and fungi [4,5]. These organisms are transmitted by direct or indirect contact with water or by inhalation of aerosol generated by a water source [6,7,8]. *Legionella* spp. are ubiquitous aquatic organisms associated with community-acquired pneumoniae as well as hospital-acquired pneumonia. Direct inhalation of aerosols from environmental colonization is typically the source of infection. As *Legionella* infection is not spread between humans, environmental monitoring of potable water, cooling towers, and related sources is crucial to control the incidence of disease. *Legionella* is able to survive for long periods in water and even to replicate in the presence of disinfectants and some conditions (e.g., pipeline materials, stagnation and sludge formation, parasitism of amoebas and protozoic cysts, and so on) [9].

In recent years, the increasing incidence of both nosocomial and community-acquired *Legionella* infections has been a major public health concern: in 2018, 2964 cases were notified to the National Surveillance System in Italy, with an incidence of 48.9 cases per million inhabitants with lethality rate for community and healthcare cases of 10.9% and 51.7%, respectively [10].

The risk of illness increases dramatically if the germ is found in certain wards such as ICUs, hematology-oncology units, cardiology units, hemodialysis units, and pulmonology units due to the critical nature of these wards for their hospitalized patients [11]. Nevertheless, the real risk of other sources of infection remains partially underestimated when making a correct *Legionella* risk assessment plan in water systems, such as suggested by the Italian Guidelines as the correct strategy to minimize the risk of colonization [12].

Different guidelines and studies have suggested that water outlets for handwashing in hospitals are frequently contaminated with *P. aeruginosa* and other Gram-negative bacteria, such as *Legionella*, which have been linked to nosocomial infections [8,13]. In particular, the presence of *Legionella* in outlets poses a risk of infection during handwashing practices due to aerosol generation.

The key factors for prevention of HAIs in the surgical area are associated with hand hygiene, surgeon handwashing characteristics, and appropriately timed glove use. Hand hygiene is an extremely important measure implemented to reduce HAIs; the WHO published guidelines in 2006 and in 2009 for routine and surgical hand hygiene protocols directed to control resident flora as well as transient microflora [14,15]. There are two primary methods for hand hygiene: antimicrobial or non-antimicrobial soap and water scrub, called the “scrub method”, and Alcohol-Based Hand Rub, called the “rub method” [16]. Concerning the surgeon handwashing station characteristics, they are generally made entirely of stainless steel with a tank made of a single plate to guarantee the continuity of the surfaces and to avoid all possible areas of bacterial proliferation (e.g., spaces or grooves). The front part is slanted by 30° in order to prevent splashing and direct water contact with operators [17].

Moreover, surgical handwashing points are equipped with two main types of faucets: manual faucets, with a long clinical lever that dispenses and mixes water by use of the elbow or foot to avoid direct contact with the hands, or non-touch water taps, provided with photocell-operated water supply as electronically managed by a photocell sensor, some of them provided by Thermostatic Mixer Valves (TMVs) [18].

Non-touch water taps, also called sensor-activated faucets with TMVs, have been gradually introduced into private and public hospital facilities to prevent patients or HCS from risk of acquiring infection or transferring infection during surgical procedures by touching contaminated taps. These taps work only when the hands are put in front of a magnetic/sensor valve which causes water to flow out and, when hands are removed, the water flow to stop. The presence of a TMV permits the flushing of water through a single pipeline at a fixed temperature (generally about 36 °C). The mixing is due to the presence of a cartridge which is able to recall cold water, leading to the desired temperature when mixed with hot water. Hospitals and other healthcare facilities where hygienic measures are very important have started to install this type of touch-free tap system to promote lower water consumption, thus saving costs and preventing HCS from potential recontamination upon hand contact with faucet valves [18]. However, there are no current data that support a decrease in HAIs associated with the use of non-touch water taps [19].

Periodic monitoring of the presence of *Legionella* or other waterborne pathogens in all outlets used for hand hygiene—in particular, during the preoperative phases of hand hygiene in surgeons—represents a preventive measure to avoid handwashing contamination before starting surgical procedures and to control the possible exposure of patients and health professionals.

Our research is presented as the result of a *Legionella* environmental monitoring program, conducted from 2013 to 2019 in 11 hospitals located in different regions of Italy. The analysis of data has identified, as critical points, 52 Surgical Handwashing Outlets (SHWOs) provided by sensor-activated faucets with TMVs with high levels of *Legionella* contamination.

The focus of the study is the analysis of microbiological contamination of SHWOs concerning *Legionella* and *P. aeruginosa*, comparing hot- and cold-water samples supplied by a municipal distribution system. The data obtained are also studied in relation to the SHWO temperatures measured as well as compare the SHWOs technologies—sensor-activated faucets with TMVs versus manual clinical valves without TMVs—to understand the key elements of contamination that could develop a reservoir for *Legionella* and could enhance the risk of infection.

## 2. Results

All results are presented, first of all, by considering the general contamination found in SHWOs and, then later, by dividing *Legionella* contamination between hot- and cold-water samples. The data about *Legionella* concentration are expressed in Log_10_ cfu/L (Log cfu/L).

The same method is used to correlate the microbial contamination found with temperature values measured in SHWOs and their distribution between hot- and cold-water samples.

### 2.1. Legionella Contamination in SHWOs

**Figure 1:**
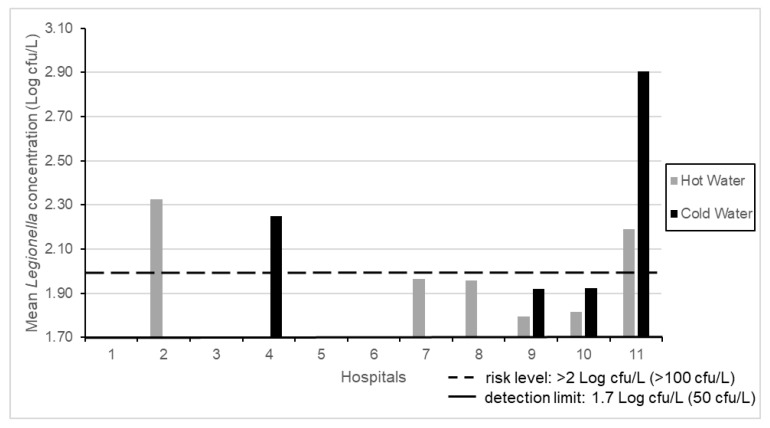
Mean *Legionella* concentrations in 11 hospitals.

The results of mean *Legionella* concentrations found in 52 SHWOs from 11 hospitals are shown in Figure 1. Seven of the hospitals showed *Legionella* contamination (7/11, 63.6%), where three (3/7, 42.8%) of them showed values over the level of risk indicated by Italian Guidelines, that is, at >100 cfu/L (>2 Log cfu/L) [12]. The contamination was found in hot or cold samples and in both water distribution systems for each hospital.

The results of microbial contamination from 669 SHWO samples show that *Legionella* was detected in 293/669 (43.8%) of samples.

An analysis of *Legionella* contamination was then performed between hot-water (*n* = 427) and cold-water samples (*n* = 242). The differences between the numbers of hot- and cold-water samples were linked to a higher concentration of *Legionella* found in hot-water samples which, according to the suggestions of the Italian Guidelines, requires resampling from the same positive outlets [12].

In particular, the analysis of results between hot- and cold-water distribution systems showed 190/427 (44.5%) of positive hot-water samples and 103/242 (42.6%) positive cold-water samples. The positive samples over the *Legionella* level of risk (>2 Log cfu/L) were 140/190 (73.7%) for hot- and 70/103 (68.0%) for cold-water samples.

In Table 1, the data of mean temperature and disinfectant residue with relative minimum (min) and maximum (max) values, the percentage of *Legionella* positive samples, mean concentrations, and the range of contamination (min–max) found in hot and cold-water samples are listed, respectively. Data about temperature, disinfectant residues, and *Legionella* concentration are expressed as mean ± Standard Deviation (SD).

No significant difference (*p* = 0.34) is found between hot and cold samples concerning *Legionella* levels.

Regarding the *Legionella* isolates distribution in SHWOs between hot- and cold-positive samples, the results showed samples contaminated only by *Legionella pneumophila (L. pneumophila)*, samples contaminated only by *Legionella* non-*pneumophila* species (other *Legionella* spp.) and others contaminated by both species. Significant differences (*p* = 0.001), obtained with the statistical χ^2^ test, were found concerning the *Legionella* spp. distribution between hot and cold samples as follows: in hot-water samples, the main isolate belonged to *L. pneumophila* 123/190 (64.7%), followed by samples with both species (*L. pneumophila* and other *Legionella* spp.) 41/190 (21.6%) and, finally, by 26/190 (13.7%) showing only the presence of other *Legionella* spp. In cold-water samples, we found the same trend, with 44/103 (42.7%) of samples with *L. pneumophila*, 30/103 (29.1%) contaminated by both species, and finally, 29/103 (28.1%) with only other *Legionella* spp. The isolates of *L. pneumophila* were identified by an agglutination test as belonging to serogroups 1, 3, 4, 6, and 8. The typing of *Legionella* non-*pneumophila* species by *mip* gene sequencing, indicated the presence of *Legionella anisa* (*L. anisa)*, *Legionella rubrilucens (L. rubrilucens), Legionella tauriniensis (L. tauriniensis), Legionella nautarum (L. nautarum),* and *Legionella steelei (L. steelei).*

The study of *Legionella* isolates in terms of mean concentration ± standard deviation (Log cfu/L ± SD) between hot- and cold-positive samples is presented in Table 2. Multiple comparisons were performed between isolates found in hot- and cold-water samples (horizontal lines), while the comparison between hot- and cold-water samples for each type of *Legionella* isolate is shown in the columns. High *L. pneumophila* concentrations were found in hot-water samples (2.92 ± 1.08 Log cfu/L) with significant difference compared to samples colonized by only other *Legionella* spp. (*p* = 0.03) and with respect to cold-water samples (*p* = 0.008). In cold-water samples, despite a high other *Legionella* spp. mean concentration (2.47 ± 0.72 Log cfu/L), a significant difference was found only with respect to samples colonized by both species (*p* = 0.0046).

### 2.2. Legionella Contamination in Relation to Water Temperature

Regarding the temperature measured between hot and cold samples, we found a range between 21.9–60.1 °C (mean value of 47.7 °C) and a range between 9.2–44.7 °C (mean value of 19.1 °C) for hot and cold samples, respectively.

The *Legionella* contamination found considering all SHWOs samples was distributed in four ranges of temperature, which were linked to relevant considerations about the environment of *Legionella* as follows:

- the first range, called “I”, represents the samples collocated at temperature values < 21 °C. This temperature range corresponds to the standard one for drinking water for human consumption;
- the second range, called “II”, was 21–45 °C, corresponding to the mixed water produced by outlets provided by TMVs;
- the third range, called “III”, corresponds to the range between 45.1–49.6 °C. This range represents the setting temperature generally measured during environmental monitoring on hot-water system producers (e.g., boilers, electric tanks, heater-exchangers, and so on), other than the values suggested to reduce energy costs [20]; and
- the fourth range, called “IV”, corresponds to temperature values > 49.6 °C. This is the optimal value suggested by the Italian Guidelines to control *Legionella* proliferation in water-distribution systems.

A multiple comparison was performed between each range by an ANOVA test, showing significant differences, as indicated in Table 3 with the (*) symbol.

The contamination of samples in relation to the temperature measured during the sampling reveals that the main *Legionella* positive samples (47.1%) were in the third range (III), which was also the main contaminated source in terms of *Legionella* mean concentration (2.17 Log cfu/L). By contrast, the lowest percentage of positive samples (23.2%) and mean concentration (1.64 Log cfu/L) were found in the fourth range (IV).

**Figure 2:**
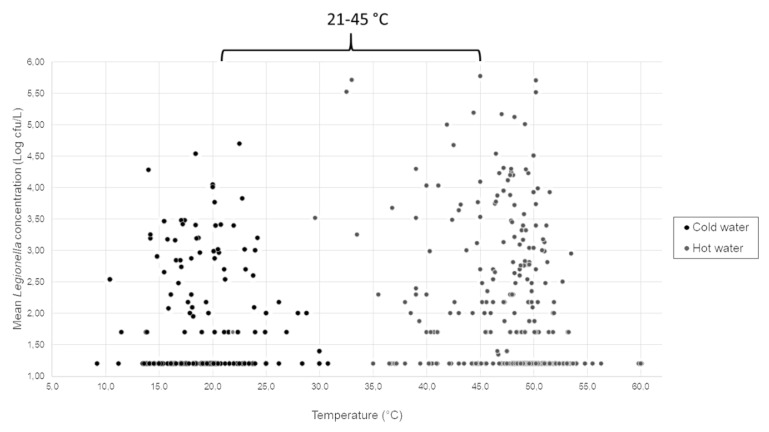
Mean *Legionella* concentration distribution in relation to water sample temperatures measured (°C).

In Figure 2, the distribution of mean *Legionella* concentration in relation to temperature values measured is represented, with hot and cold samples separately considered, in ranges between 21.9–60.1 °C (mean value of 47.7 °C) and between 9.2–44.7 °C (mean value of 19.1 °C).

An analysis of the results considering only samples in the range of 21–45 °C (e.g., the range for SHWO mixed water) showed 98/427 (23.0%) and 81/242 (33.5%) contaminated hot- and cold-water samples, with mean concentrations of 2.12 ± 1.22 Log cfu/L and 1.87 ± 0.92 Log cfu/L, respectively.

Considering only *Legionella*-positive samples, we found 52/98 (53.0%) in hot water—respectively 39/81 (48.1%) in cold water—with mean *Legionella* concentration higher in hot (2.94 ± 1.17 Log cfu/L) than cold samples (2.60 ± 0.87 Log cfu/L). The nonsignificant difference was found using the Mann–Whitney test (*p* = 0.22).

### 2.3. Legionella Contamination before and after the SHWO Replacement

In three hospitals (called 1, 8, and 11), following renovation works, replacement of sensor-activated faucets with TMVs by clinical valves without TMVs was carried out. The reassessment of *Legionella* contamination on the same SHWOs after replacement permitted us to observe changes in the *Legionella* concentration. Analyzing the contamination found in 110 of 669 total samples collected in these hospitals, we compared the contamination before (*n* = 55) and after (*n* = 55) replacement. As shown in Table 4, we observed a significant decrease in terms of *Legionella* contamination (*p* = 0.001) with the same significant trend in each hospital, other than with an increase of hot-water temperature and a consequent decrease of *Legionella* levels.

### 2.4. P. aeruginosa Contamination in SHWOs

The data about *P. aeruginosa* contamination indicated that 27/669 (4.0%) samples were contaminated. Considering the contamination in relation to hot- and cold-water circuits, we found a higher contamination in cold-water samples compared to hot-water samples: 22/242 (9.0%) and 5/427 (1.2%), respectively. However, the low number of positive samples did not permit us to find a statistical correlation between the data analyzed (*p* = 0.65).

### 2.5. Disinfectant Residue Analysis

Concerning the disinfectant residue measured, the mean concentration of hydrogen peroxide (H_2_O_2_) component was about 2.5 mg/L and 10 mg/L in cold- and hot-water samples, respectively. Although only the hot water network is treated with hydrogen peroxide/Ag^+^ (H_2_O_2_/Ag^+^), we found the presence of disinfectant residue in all cold-water samples, with a range between 0.5–5 mg/L.

## 3. Discussion

The prevention of HAIs is an important problem, particularly in high-risk patient care. The risk of infections has been linked to interactions between pathogens and hosts which involves the number of microorganisms, their virulence factors, and the host’s immune defenses [21]. To reduce the impact on human health as well as to avoid economic, legal, and political issues, particular attention must be directed to a hospital’s hygiene and environment. This aim can arise only through the development of a risk assessment plan which is linked to knowledge of the hospital and patient characteristics, the health-care procedures already in place and to be improved, and the hospital environment where patients and HCS may be in contact with microorganisms through the air, water, and contaminated surfaces [6,22,23].

The new revision of the European Drinking Water Directive, such as the WHO Guidelines for Drinking Water Quality, suggests the approach of the Water Safety Plan to identify the main pathogens involved in waterborne diseases, to understand their pathogenic pathways, and to contain their impact on public health [24,25,26,27,28]. *Legionella* and *P. aeruginosa* are two of the main waterborne pathogens involved in hospital environments associated with nosocomial infections [6,29,30].

This study reports knowledge acquired during a *Legionella* environmental surveillance program performed in hospitals, where high *Legionella* levels were detected in SHWOs with TMVs, some of them with concentrations over the risk level (>2 Log cfu/L), suggesting their critical role in bacterial growth and HAI risk. It has been well documented that temperature is a key factor in microbial growth and that, in particular, the mixing of hot and cold water creates an optimal temperature for bacterial environment, which can occur in SHWOs [8,23,31,32].

To analyze the contamination found in SHWOs, hot- and cold-water data sets were separately studied in terms of percentage of positive samples, level of contamination, and *Legionella* isolates distribution, including temperature as a possible determining factor for data fluctuations in the microbial parameters analyzed.

The results showed a similar percentage of hot- and cold-water samples (44.5% and 42.6%, respectively) contaminated by *Legionella*, with the same trend regarding samples over the *Legionella* risk level (73.7% hot vs. 68.0% cold). A nonsignificant difference in terms of *Legionella* contamination between hot and cold samples (*p* = 0.34) demonstrates how hot- and cold-water circuits are not separate with continuous mixing between two pipelines, creating an environment capable of supporting *Legionella* growth.

These results are supported by the residues of H_2_O_2_ disinfectant found also in cold-water samples. This disinfectant introduced in hospitals is injected only into the return line of the hot-water distribution system, and generally, when the two main distribution systems (e.g., hot and cold) are well separated, the cold water is expected to be free of disinfectant residues. This observation can be attributed to damage on the TMV cartridge because, during cold-water sampling, although the TMVs were deactivated, we found disinfectant residues in all samples. Moreover, damage in the TMV device was supported by the temperatures measured, which revealed a decrease in hot-water values and an increase in cold-water values, as demonstrated by the large ranges of temperature: 21.9–60.1 °C and 9.2–44.7 °C for hot and cold, respectively.

Considering the distribution of *Legionella* isolates, a significant difference was found between hot- and cold-water-positive samples (*p* = 0.001), showing that the characteristics of the mixed water produced are able to influence the distribution of isolates. According to knowledge about *Legionella* ecology and epidemiological data, the main positive samples found in hot and cold water (64.7% vs. 42.7%) belonged to *L. pneumophila* (serogroups 1, 3, 4, 6, and 8). In a low percentage of hot- and cold-water samples, we found isolates belonging to *Legionella* non-*pneumophila* species (*L. anisa*, *L. rubrilucens*, *L. tauriniensis*, *L. nautarum*, and *L. steelei*), with high values in cold water compared to hot water (28.1% vs. 13.7%). The same differences were found between cold and hot samples regarding the percentage of positive samples contaminated by both species (*L. pneumophila* and other *Legionella* spp.).

These data required supplementary analysis regarding the level of contamination found inside each distribution system and between them. In hot-water samples, we found a higher *Legionella* contamination in samples contaminated by both isolates, with a significant difference with respect to the level of contamination found in samples with only other *Legionella* spp. (*p* = 0.00012). A significant difference was, therefore, found in terms of the level of contamination between *L. pneumophila* and other *Legionella* spp. (*p* = 0.03).

In cold-water samples, we observed a different trend, with high samples contaminated by both species showing significant differences with respect to samples having only *L. pneumophila* (*p* = 0.0012) and samples contaminated by only other *Legionella* spp. (*p* = 0.0046).

Considering the comparison of mean concentration found for each isolate between hot versus cold samples, a significant difference was found only for *L. pneumophila* (*p* = 0.008).

Relevant information comes from these results regarding the ecology of isolates in water distribution systems.

*Legionella* lives in a water environment, with optimal growth in warm environments. Therefore, the abundance of *L. pneumophila* in hot-water samples found was in line with data about the high incidence of this species in human disease. In hot-water environments, there is likely a selective pressure of *L. pneumophila* on *Legionella* non-*pneumophila* species, which is suppressed in cold-water distribution systems, as demonstrated by the high number of samples with both species when the water temperature was mixed. Our hypothesis is also based on observations done during *Legionella* culture, where we generally find a lower *Legionella* non-*pneumophila* species isolation rate, due to their slow growth and late detection after 10–15 days of incubation when *L. pneumophila* is more abundant. When the culture technique was conducted up to 10 days, some of these species were missing and, consequently, underestimated; by contrast, an extension of culture timing permits their detection.

The poor awareness of these species and their underestimation is also associated to the low rate of clinical isolation, to their low correlation with human disease, and to the non-detection by diagnostic techniques (e.g., antigenic urinary tests) [33,34].

Another important point that can explain the high presence of *Legionella* non-*pneumophila* species in cold water is related to the disinfection treatment that often, as seen in this study, is performed on the hot-water circuit, leaving the cold-water distribution system without any type of control (monitoring by culture, temperature measures, flushing, and disinfectant residues measures). This represents a reservoir for other *Legionella* species. The absence of disinfectant or low levels of disinfectant residues measured usually require high temperatures for their activation and are unable to control their growth.

These results were also confirmed by our previous data [35] regarding the ability of *Legionella* to colonize and increase its concentration in cold-water distribution systems, inducing a change of cold water microflora; during renovation works, pipeline, TMV, and faucet damage; or when rapid breakdown of hot temperatures occurs. The presence of a high percentage of positive samples with high *Legionella* concentration contaminated by both species in both distribution systems confirms that SHWOs with mixed water develop an environment favorable to *Legionella* growth.

The high contamination of SHWOs are therefore supported by a wide fluctuation of temperatures found in samples: both low and high temperatures are able to favor bacteria growth. The analysis of contamination levels with respect to temperatures was analyzed by dividing the temperature values measured between four ranges, each of them associated to the ecology of *Legionella*.

The possibility to maintain separation between cold- and hot-water pipelines is one of the strategies suggested by National and European directives in order to contain the proliferation of bacteria. Our data demonstrated an inverse correlation between the temperature and bacteria load: at higher temperatures (>49.6 °C), a lower *Legionella* mean concentration (1.64 Log cfu/L) was observed, according with the directive’s suggestions about the value of >50 °C being able to perform complete control of the level of *Legionella* [12].

The results obtained inside the II and III ranges of temperature (21–45 °C and 45.1–49.6 °C, respectively) showed approximately the same *Legionella* concentrations with a nonsignificant difference inside these ranges (*p* = 0.474). These data confirm that samples with temperature close to the optimum *Legionella* growth range (25–42 °C) are more contaminated and that an increase of temperature (>49.6 °C) leads to control of the *Legionella* proliferation (II vs. IV, *p* = 0.012; III vs. IV, *p* = 0.001) [36].

The contamination in SHWOs and the wide range of temperatures found can be explained, moreover, by taking into account the SHWO technology provided by hospitals. All of them are characterized by the presence of magnetic valves, which are the principal part of electronic/non-touch/sensor tap systems. Cold and hot water from the junctions of the central water pipeline system are mixed to provide an acceptable and comfortable setting temperature, generally, around 36 °C. The magnetic valves in the cartridge are made of material membranes—for example, made of rubber, plastic, or Polyvinyl Chloride (PVC)—which are very hard to disinfect and easily enhance bacterial growth and biofilm development, which can become a protective envelope against biocides and disinfectants. Furthermore, in these tap systems, flushing procedures are forbidden by the presence of a photocell system, leading to low water pressure and flow [18]. These considerations were supported by data about positive samples located in the range of the TMVs’ working temperature (21–45 °C), which is very close to the temperature associated with optimal *Legionella* growth, where we did not find a difference between hot and cold samples.

Our hypothesis is strengthened furthermore by the observation that, in three hospitals which implemented a substitution program from sensor-activated faucets with TMVs to manual clinical valves without TMVs, an increase of mean temperature was measured, corresponding to a significant reduction trend in *Legionella* concentration levels.

As concerning *P. aeruginosa* SHWO contamination, the lower presence of positive samples coming from the eleven hospitals suggests the general good performance of disinfection procedures applied by hospital staff on faucets. The choice of tapware provided by faucet aerators guarantees low pressure without an internal thread, and descaling and disinfection procedures are applied weekly, permitting to avoid bacterial growth on outlets and preventing biofilm development [32].

The data regarding the higher *P. aeruginosa* contamination in cold-water samples can be explained by the same consideration as for sensor-activated faucets in *Legionella* contamination due to the sharing of these bacteria in the same habitat.

These findings led to the following considerations:

- the implementation of environmental monitoring in the cold-water distribution system, where *Legionella* surveillance is often missing, helps to explain the lower hot-water temperature sometimes observed also in hot water, which is often associated to damage in the mixing water system (e.g., in TMVs, levers, or faucets);
- the replacement of broken devices avoids the necessity of use of disinfection treatment in the whole distribution system, which can enhance bacterial resistance according to Berjeaud et al. [37]; and
- the continuous mixing between hot and cold water produced by TMVs leads to a mixture regarding the distribution of *Legionella* isolates in hot- and cold-water systems, as suggested by our data, developing a potential source of infection in cold water.

## 4. Materials and Methods

The eleven hospitals of this study, numbered 1 to 11, were involved in a *Legionella* environmental surveillance program from 2013 to 2019. After the introduction of last version of the Italian Guidelines in 2015, the 11 hospitals developed a risk assessment plan for *Legionella* control, considering the locations of buildings, their types of patients, and the water distribution system characteristics. All hospitals were supplied by municipal water that, after softener treatment, was heated by a heat-exchanger along with a hot-water return line.

All hospitals performed a six-month plan of *Legionella* environmental monitoring and active surveillance to control nosocomial *Legionella* infection by urinary-antigen test. Therefore, a complete program of maintenance procedures by measuring and recording temperatures, flushing outlet points, continuous disinfection of the system by H_2_O_2_/Ag^+^, and a fortnightly plan regarding aerator disinfection and/or replacement was undertaken.

During environmental monitoring, we found a higher *Legionella* concentration in SHWOs with respect to other hospital outlets involved in monitoring, indicating the necessity of a supplementary investigation.

**Figure 3:**
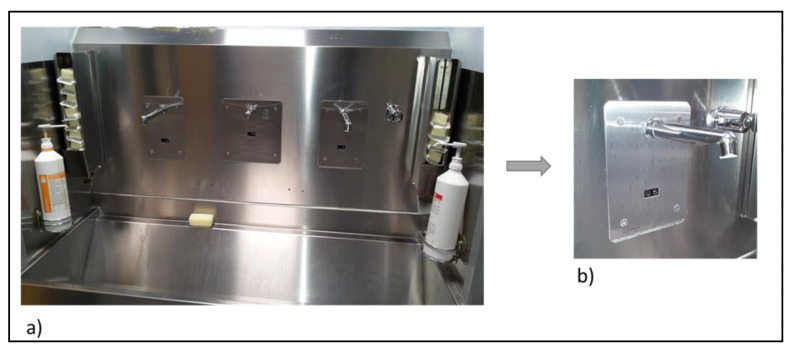
SHWOs with sensor-activated faucets and TMVs (**a**) and a sensor-activated faucet (**b**).

In Table 5, the number of SHWOs (*n* = 52) in each hospital is reported, all of them equipped with sensor-activated faucets with TMVs (Figure 3). The main distribution system supplied hot-water outlets in a temperature range between 40–50 °C, while the cold-water outlets showed a temperature range of 15–20 °C. In SHWOs, the presence of TMVs produced a continuous mixed water at a set temperature around 36 °C.

**Figure 4:**
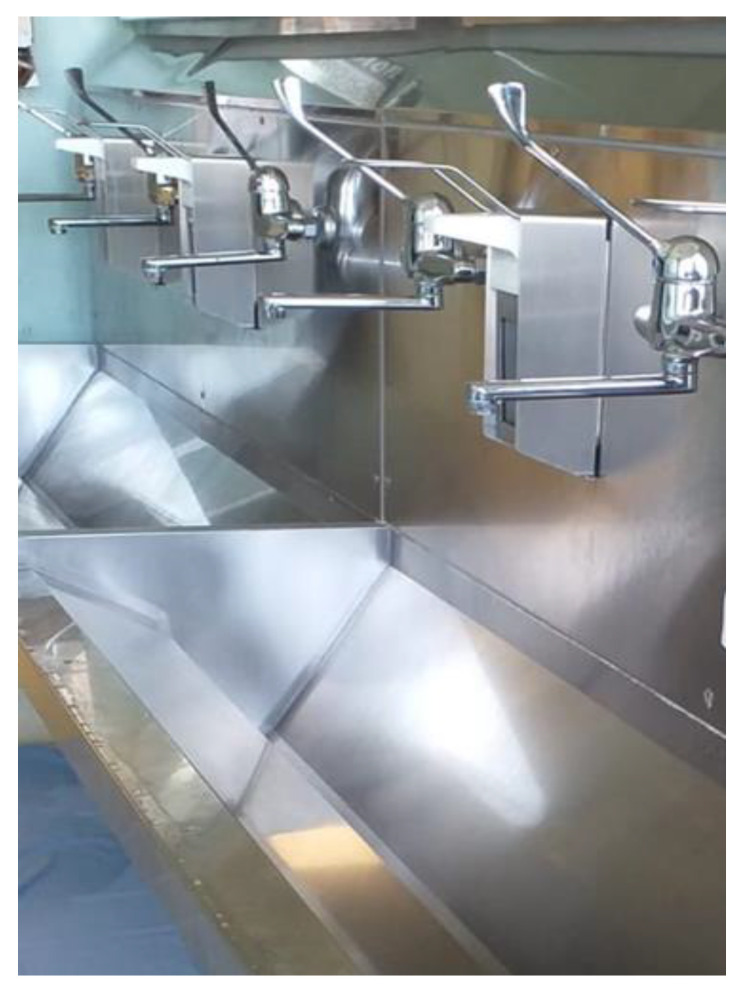
SHWOs with elbow-operated manual faucets without TMVs.

During the study, eight hospitals had not implemented any replacement in SHWOs; however, three hospitals (1, 8, and 11) implemented a substitution program for their surgical hand preparation points regarding the faucet apparatuses: sensor-activated faucets with TMVs were removed and substituted with elbow-operated manual faucets without TMVs (Figure 4).

The environmental surveillance program consisted of *Legionella* and *P. aeruginosa* monitoring, according to the risk assessment plans provided by hospital healthcare directives.

The hot-water circuit in all hospitals was treated by H_2_O_2_/Ag^+^ disinfectant, which was added by a pump proportionally to the volume of cold water supply at a concentration around 50 mg/L in order to allow a residue at outlets between 10–20 mg/L, following manufacturer’s instructions.

To assess the complete monitoring of water microbiological quality supplied by SHWOs and to evaluate differences in terms of contamination between hot- and cold-water distribution systems, both circuits were sampled.

For the three hospitals that implemented a substitution program with manual clinical valves without TMVs, the data of cold-water samples were not available, as the risk assessment plan after replacement involved only hot SHWO samples; therefore, comparison in terms of *Legionella* contamination before and after the substitution program was considered only in hot-water circuits.

### 4.1. SHWO Sampling

According to the Italian Guidelines for the Prevention and Control of Legionellosis [12], analysis of *Legionella* contamination was performed by collecting two liters of cold and hot SHWO samples. In particular, in order to determine the quality in the main distribution system, post-flushing sampling was applied, which consisted of removing the filter or faucets, disinfection of taps with ethanol (70%), open taps, flushing for 2 min, and collection of cold before hot samples [38]. For cold samples, the TMVs were deactivated; by contrast, TMVs were reactivated to collect hot samples at the setting temperature for SHWOs (36 °C).

From the 52 SHWOs, 669 samples were collected (427 hot and 242 cold), where two liters of water were sampled using 1-liter sterile polytetrafluoroethylene (PTFE) bottles containing sodium thiosulphate (20 mg/L) [38,39].

The samples were processed by a membrane-filtration technique using polyethersulfone membrane filters with a porosity of 0.22 μm (Sartorius, Bedford, MA, USA), according to the International Standard Organization (ISO) 11731:2017 procedure [40].

### 4.2. Legionella and P. aeruginosa Culture and Typing

The *Legionella* culture was performed on Glycine-Polymyxin B-Vancomycin-Cycloheximide (GVPC) plates (Thermo Fisher Diagnostic, Basingstoke, UK) and subsequently incubated at 36 ± 1 °C with 2.5% CO_2_. *Legionella* growth was evaluated every 2 days for a total of 15 days of culture.

After the incubation period, the colonies with morphologies associated to the *Legionella* genus were enumerated and five suspected colonies for each morphology, as indicated by ISO 11731:2017, were subcultured on Buffered Charcoal Yeast Extract (BCYE) agar with l-cysteine (cys+) and without l-cysteine (cys−) as supplement, which is a selective media used for *Legionella* isolation. The positive *Legionella* colonies were those that grew on *Legionella* BCYE cys+ agar but failed to grow on *Legionella* BCYE cys− agar.

The isolates grown on BCYE cys+ were serologically typed by an agglutination test (*Legionella* latex test kit, Thermo Fisher Diagnostic, Basingstoke, UK). The isolates identified as *L. pneumophila* were then processed for serogroup identification by polyclonal latex reagents (Biolife, Milan, Italy).

Colonies identified by the agglutination test as belonging to *Legionella* non-*pneumophila* species were subsequently analyzed by *mip* gene sequencing and by Polymerase Chain Reaction (PCR) using degenerate primers (as described by Ratcliff et al. [41]) and modified by M13 tailing to avoid noise in the DNA sequence [42]. Gene amplification was carried out in a 50-μL reaction containing DreamTaq Green PCR Master Mix 2× (Thermo Fisher Diagnostic) and 40 picomoles of each primer; 100 nanograms of DNA extracted from the presumptive colonies of *Legionella* was added as a template. The same amounts of DNA from *L. pneumophila* type strain EUL00137, provided by the European Working Group for *Legionella* Infections (EWGLI) [43], and fetal bovine serum were used as positive and negative controls, respectively.

Following purification, DNA was sequenced using BigDye Chemistry and analyzed on an ABI PRISM 3100 Genetic Analyzer (Applied Biosystems, Foster City, CA, USA). Specifically, *mip* amplicons (661–715 base pairs) were sequenced using M13 forward and reverse primers (mip-595R-M13R caggaaacagctatgaccCATATGCAAGACCTGAGGGAAC; mip-74F-M13F tgtaaaacgacggccagtGCTGCAACCGATGCCAC) to obtain complete coverage of the sequenced region of interest. Raw sequencing data were assembled using the CLC Main Workbench 7.6.4 software (QIAGEN, Redwood City, CA, USA). The sequences were compared with sequences deposited in the *Legionella mip* gene sequence database using a similarity analysis tool. Identification on the species level was done based on ≥98% similarity to a sequence in the database [44].

The results regarding *Legionella* contamination in the samples were expressed as colony formant unit (cfu) per liter (cfu/L). According to ISO 11731:2017, a negative result (absence of bacteria growth) was expressed as the lower limit of detection, that is, <50 cfu/L [40].

The same samples (*n* = 669) were analyzed to quantify the presence of *P. aeruginosa* due to its role in biofilm formation and to its capacity to inhibit *Legionella* growth during isolation culture, producing inaccurate results [45]. The analysis was performed on a volume of 100 mL of hot and cold samples, filtered using a cellulose nitrate membrane filter with a 0.45-μm pore size (Sartorius, Bedford, MA, USA), according to UNI EN ISO 16266:2008 [46,47].

The membrane was seeded on *Pseudomonas*-selective agar plate (PSA, Biolife, Milan, Italy) and incubated for 48 h in 36 °C incubators. Colonies that showed green-blue fluorescence when placed under a Wood’s lamp (ultraviolet light at 365 nm) were subcultured on Nutrient agar (NA, Biolife, Milan, Italy) for 18–24 h. Subsequently, the colonies were identified biochemically as *P. aeruginosa* by indole, oxidase reaction tests, and BBL Crystal Enteric/Non Fermenter ID Kit (Becton Dickinson Systems, Cockeysville, MD, USA), according to the manufacturer’s instructions.

The results are expressed in terms of cfu/100 mL.

### 4.3. Physical and Chemical Analyses

The physical and chemical parameters—the temperature of the water samples as well as the disinfectant residues at SHWOs—were measured during the collection of samples.

The temperature (°C) (T) was measured by a conductivity meter coupled with a thermistor probe (Temp 6 basic for probe Pt100 RTD from −50 to +199 °C; Thermo Fisher Scientific Inc., Eutech Instruments Pte Ltd., Singapore). An on-site commercial kit for the residual hydrogen peroxide component of H_2_O_2_/Ag^+^ (mg/L) was used. The kit uses a colorimetric test based on peroxidase activity to transfer peroxide oxygen to an organic redox indicator, which produces a blue oxidation product.

The hydrogen peroxide concentration was measured semiquantitatively by visual comparison of the result seen on the reaction zone of the test strip with the fields on a color scale in a range of 0.5–25 mg/L H_2_O_2_.

### 4.4. Statistical Analysis

The *Legionella* concentration data were converted into Log_10_ cfu/L (Log cfu/L) to normalize the non normal distributions. According to the Italian Guidelines for *Legionella,* the detection limit corresponding to 50 cfu/L (1.7 Log cfu/L) was used; by contrast, the risk value, >100 cfu/L, was expressed as >2 Log cfu/L.

To compare data of hot- and cold-water *Legionella* concentrations, the Mann–Whitney test was used (Table 1).

The distribution of different *Legionella* isolates between hot and cold positive samples was studied by chi-squared test (χ^2^). Therefore, the differences in *Legionella* isolate concentrations in hot- or cold-water samples were studied by the Kruskal–Wallis test: the significant data found were then also analyzed by the Mann–Whitney test (Table 2).

Multiple comparisons between *Legionella* concentrations and the four ranges of temperature measured were performed by using the ANOVA test (Table 3).

Regarding the three hospitals that implemented the replacement program (e.g., with elbow-operated clinical valves without TMVs), the data analysis to compare *Legionella* levels before and after replacement was performed by parametric *t*-Student test when considering a number of values *n* > 30 and by nonparametric Wilcoxon test for *n* < 30 (Table 4).

The *P. aeruginosa* results were converted into Log cfu/100mL. The contamination found was studied by Mann–Whitney test to compare hot- and cold-water samples.

Statistical analyses were performed using the SPSS software for Windows version 23 (IBM SPSS, Inc., Chicago, IL, USA).

The data were considered significant for *p* values (*p*) ≤ 0.05.

## 5. Conclusions

In conclusion, sensor-activated faucets with TMVs are generally more contaminated than clinical valves without thermostatic mixers. This allows us to conclude that the technologies typically chosen by a hospital do not correspond with the water microbiological environment that can develop in the SHWOs. The microbial interaction with the selected technologies, pipeline and faucet materials, and chemical-physical water characteristics result in an environment that, in semi-critical and critical areas, can lead to serious risks for patients, hospital staff, and stakeholders involved in maintenance procedures. The limit of this study is the lack of data on cold water after the replacement program developed by three hospitals due to there being no cold-water monitoring in the risk assessment plan, to poor knowledge, and to cost-containment demands.

The authors wish to encourage infection control teams to evaluate the use of non-touch fittings in hospitals, especially when installed in high-risk areas, and wish to promote water microbial monitoring in both hot- and cold-water distribution systems according to a water safety plan that can guide the hospital’s choices based on epidemiological data, technological knowledge, and applied maintenance procedures.

## Figures and Tables

**Table 1:** Surgical Handwashing Outlet (SHWO) microbiological and physical-chemical parameters measured: hot- vs. cold-water samples.

SHWO Distribution Systems	Temperature Mean ± SD (Min–Max) (°C)	H_2_O_2_ Residue Mean ± SD (Min–Max) (mg/L)	Number of Total SHWO Water Samples	Number of *Legionella*-Positive Samples/Total Samples (%)	*Legionella* Concentration Mean ± SD (Min–Max) (Log cfu/L)	Mean *Legionella* Concentration Comparison Hot vs. Cold Samples Mann–Whitney Test *p*-Value (*p*)
Hot water samples	47.7 ± 4.95 (21.9–60.1)	10 ± 6.67 (5–25)	427	190/427 (44.5)	1.94 ± 1.07 (1.70–5.8)	0.34
Cold water samples	19.1 ± 4.38 (9.2–44.7)	2.5 ± 1.5 (0.5–5)	242	103/242 (42.6)	1.81 ± 0.88 (1.70–4.7)	

**Table 2:** *Legionella* isolate mean concentration comparison in SHWOs: hot- vs. cold-water samples.

*Legionella* Isolate	Samples with Only *L. pneumophila* Mean ± SD (Log cfu/L)	Samples with Only Other *Legionella* spp. Mean ± SD (Log cfu/L)	Samples with *L. pneumophila* and Other *Legionella* spp. Mean ± SD (Log cfu/L)	*Legionella* Isolate Mean Comparison in Hot and Cold Water Mann–Whitney Test *p*-Value (*p*)	
Hot water samples	2.92 ± 1.08	2.31 ± 0.66	3.13 ± 0.85	*L. pneumophila* vs. other *Legionella* spp.	0.03 *
				*L. pneumophila* vs. *L. pneumophila* and other *Legionella* spp.	0.40
				other *Legionella* spp. vs. *L. pneumophila* and other *Legionella* spp.	0.00012 *
Cold water samples	2.43 ± 0.83	2.47 ± 0.72	3.09 ± 0.63	*L. pneumophila* vs. other *Legionella* spp.	1.00
				*L. pneumophila* vs. *L. pneumophila* and other *Legionella* spp.	0.0012 *
				other *Legionella* spp. vs. *L. pneumophila* and other *Legionella* spp.	0.0046 *
*Legionella* Isolate Mean Comparison between hot vs. cold samples Mann–Whitney test *p*-value (*p*)	0.008 *	0.4	0.7		

* Values are statistically significant at *p* ≤ 0.05.

**Table 3:** Mean *Legionella* concentration in relation to ranges of temperature measured (I, II, III, and IV).

Range of Temperature (°C)		Number of Samples	Number of Positive Samples (%)	Mean *Legionella* Concentration (Log cfu/L)	95% Confidence Interval (CI)	Range of Temperature Comparison	ANOVA Test *p*-Value *(p)*
I	<21	168	54 (32.1)	1.78	1.65–1.91	vs. II	0.464
						vs. III	0.002*
						vs. IV	1.000
II	21–45	157	59 (37.6)	1.98	1.81–2.15	vs. I	0.464
						vs. III	0.474
						vs. IV	0.012 *
III	45.1–49.6	172	81 (47.1)	2.17	2.00–2.34	vs. I	0.002 *
						vs. II	0.474
						vs. IV	0.001 *
IV	>49.6	172	40 (23.2)	1.64	1.51–1.78	vs. I	1.000
						vs. II	0.012 *
						vs. III	0.001 *

* Values are statistically significant at *p* ≤ 0.05.

**Table 4:** Mean *Legionella* concentration in three hospitals before and after the replacement of sensor-activated faucets with Thermostatic Mixer Valves (TMVs).

ID Hospitals	Number of SHWOs (Total Samples)	Time of Renovation Works of SHWOs (Total Samples)	Mean Temperature Samples (°C)	Number of *Legionella* Positive Samples/Total of Samples (%)	Number of *Legionella* Samples Over Risk Value/Positive Samples (%)	Mean *Legionella* Concentration ± SD (Log cfu/L)	t-Student and Wilcoxon Test *p*-Value (*p*)
1	5 (28)	Before (14)	42.73			1.98 ± 1.34	0.046 *
				5/28 (17.9)	4/5 (80.0)		
	
		After (14)	49.15			1.23 ± 0.13	
				1/28 (3.6)	0		
	
8	3 (50)	Before (25)	42.89			2.59 ± 1.34	0.001 *
				19/50 (38.0)	13/19 (68.4)		
	
		After (25)	46.98			1.3 ± 0.20	
				5/50 (10.0)	0		
	
11	14 (32)	Before (16)	48.96			3.03 ± 1.04	0.001 *
				15/32 (46.9)	15/15 (100.0)		
	
		After (16)	49.50	9/32 (28.1)	5/9 (55.6)	1.8 ± 0.86	

* Values are statistically significant at *p* ≤ 0.05.

**Table 5:** Number of SHWOs/hospitals.

**Number** **of** **SHWOs** **(*n* = 52)**	**ID Hospitals**										
	1	2	3	4	5	6	7	8	9	10	11
	5	5	5	1	6	2	2	3	4	5	14
